# Towards an objective measurement of sleep quality in non-human animals: using the horse as a model species for the creation of sleep quality indices

**DOI:** 10.1242/bio.059964

**Published:** 2023-07-20

**Authors:** Linda Greening, Sian Allen, Sebastian McBride

**Affiliations:** ^1^ Equestrian Performance Centre, Hartpury University, Gloucester GL19 3BE, UK; ^2^ Department of Life Sciences, Aberystwyth University, Ceredigion SY23 3DA, UK

**Keywords:** Sleep quality metric, Non-invasive measure, Sleep disruption, Equine

## Abstract

Sleep disturbance is observed across species, resulting in neurocognitive dysfunction, poor impulse control and poor regulation of negative emotion. Understanding animal sleep disturbance is thus important to understand how environmental factors influence animal sleep and day-to-day welfare. Self-reporting tools for sleep disturbance commonly used in human research to determine sleep quality cannot be transferred to non-verbal animal species research. Human research has, however, successfully used frequency of awakenings to create an objective measurement of sleep quality. The aim of this study was to use a novel sleep-quality scoring system for a non-human mammalian species. Five separate sleep quality indices calculations were developed, using frequency of awakenings, total sleep time and total time spent in different sleep states. These indices were applied to a pre-existing data set of equine sleep behaviour taken from a study investigating the effects of environmental change (lighting and bedding) on the duration of time in different sleep states. Significant treatment effects for index scores both differed and aligned with the original sleep quantity results, thus sleep quality may be a useful alternative measurement of sleep disturbance that could be used to investigate impactful (emotional, cognitive) effects on the animal.

## Teaser

**Summary:** Unlike in humans, the quality of animal sleep cannot be assessed via verbal subjective report. This study sought to use quantitative scoring indices to measure sleep quality in the horse (*Equus caballus*).

## INTRODUCTION

Sleep disturbance in humans produces a number of deleterious effects including neurocognitive dysfunction, poor impulse control (Fabbri et al., 2021), impaired executive functioning (Tucker et al., 2010), poor regulation of negative emotion (Mauss et al., 2013) and depression (Jakubcakova et al., 2012). Disturbed sleep has been shown to have a similar effect in animals, for example sleep disruption leads to significantly reduced interspecific emotion recognition in dogs (*Canis familiaris* – Bolló et al., 2020), and behavioural communication is affected in honey bees (*Apis mellifera* – Klein et al., 2010). Understanding and being able to measure sleep quality in different animal species is therefore paramount if we are to understand how environmental factors can affect animal sleep and thus impact their day-to-day welfare. To date, animal sleep research has largely focused on cumulative quantity relative to total sleep time, as opposed to the quality of sleep. This may be an important limitation because, in human studies, sleep quantity does not reflect the subjective sleep experience (for example, Bei et al., 2010). Although subjective reports of sleep quality in humans have previously been compared to objective measures of sleep quantity, including sleep onset latency (SOL), total wake time (TWT), and total sleep time (TST) (Mendonҫa et al., 2019), these metrics are not always capable of differentiating between different levels of sleep quality. For example, measures often do not differ significantly between normal sleepers and insomniacs or individuals who continue to subjectively report poor quality sleep (Krystal and Edinger, 2008). In contrast, other characteristics of sleep architecture have been more closely linked to subjective reports of sleep quality, primarily the number of awakenings during total sleep time (Rosipal et al., 2013).

Sleep awakenings are often referred to as sleep disturbance, disruption, fragmentation, interruption, arousal and wake sequences/episodes, with each being defined differently. For example, sleep fragmentation has been defined as an increase in electroencephalography (EEG) frequency and electromyography (EMG) amplitude lasting for more than 3 seconds (Stepanski et al., 1984). Meanwhile sleep interruptions have been defined as any sleep cycle with more than 3 minutes of continuous wakefulness for humans (Merica and Gaillard, 1986), after which the individual cannot easily revert back to sleep (Halász et al., 2004). Wake sequences in rats have also been described in similar terms but with a threshold of 300 s used to distinguish between brief and long duration wake episodes (Simasko and Mukherjee, 2009). While awakenings have some functional significance for normal sleep (Halász et al., 2004; Chaun Lo, 2004), they have also been described as markers of sleep disruption representing a detrimental and harmful feature for sleep (Atlas Task Force, 1992). The occurrence of sleep awakenings between species is variable, possibly due to the variation in the functional significance of sleep (Greening and McBride, 2022) and the differences in total sleep time (Campbell and Tobler, 1984). For example, mouse sleep is highly fragmented, with sleep episodes lasting less than 5 min (Toth and Bhargava, 2013), and rat sleep can be interrupted by a high number of wake episodes (38-40; <300 s) (Simasko and Mukherjee, 2009). Meanwhile, equine sleep episodes last on average 40.74 min (Greening and McBride, 2022) with an average of seven wake sequences (<3 min) per sleep cycle (Kalus, 2014) compared to humans which have sleep episodes lasting 80-200 min (Le Bon et al., 2002) with an average of 0.64 wake sequences per sleep cycle (Greening and McBride, 2022). These profiles of species-specific awakenings are considered to be part of the normal sleep pattern (Chaun Lo, 2004). In this context, disrupted sleep is when the number of awakenings is significantly above this normal pattern but also when these awakenings transition into a state of wakefulness that subsequently affects the duration of total sleep time. Establishing criteria for sleep interruptions and awakenings in relation to total sleep time (total sleep state time) could therefore provide a useful basis for developing objective measures of sleep quality in non-human species.

The use of subdural and surface electrodes for EEG and polysomnography (PSG) has proven successful for recording sleep in a range of animals e.g. horses (*Equus caballus* – Williams et al., 2008), dogs (*Canis familiaris* – Kis et al., 2017), and cows (*Bos taurus taurus* – Hunter et al., 2021), however there are some disadvantages to this approach. For example, the continuity and accuracy of 24 h observations can be affected by movement of superficial facial and ear musculature, loss of contact between electrode and surface of the head, and influence of sweat and other extraneous factors (Hunter et al., 2021; Zanker et al., 2021). EEG outputs are also complex and voluminous, and time consuming in their interpretation. EEG equipment is also expensive and technically demanding, and is often limited for use in non-human species. The alternative of behavioural sleep scoring has advantages and disadvantages, including human interpretation of sleep state, time required for sleep scoring and overlap in behaviours between different sleep states (Greening and McBride, 2022). In terms of developing a measurement of sleep quality in non-human animals, the methodological approach (EEG versus behaviour) will have implications on the validity of the sleep quality metric. For example, in an experimental situation where changes in the environment are used to induce changes in quantity and quality of sleep, different levels of conclusion can be drawn depending on which metrics are used (Table 1). Overall EEG metrics allow for firmer conclusions, whereas behavioural metrics can create alternative interpretations of the data. In this study, we employed a behavioural approach, for some of the reasons stated above but primarily with the aim of developing a sleep quality index (SQI) that is not reliant on specialist equipment and that can be derived from the more accessible measurements of movement and posture of the animal.

**Table 1.**
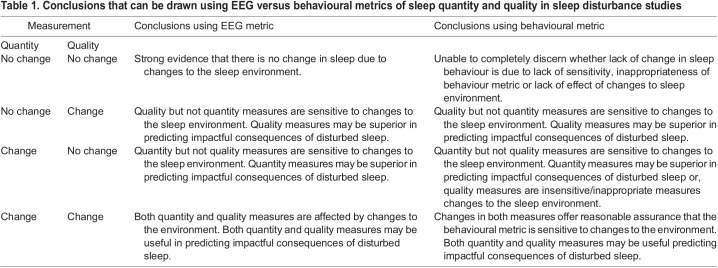
Conclusions that can be drawn using EEG versus behavioural metrics of sleep quantity and quality in sleep disturbance studies

Equine sleep has been well characterised with evidence of the occurrence of rapid eye movement (REM) and non-rapid eye movement (NREM) sleep states, and sleep fragmentation (Greening and McBride, 2022). As a prey species, the horse is typically vigilant and demonstrates high levels of wake sequences during sleep cycles. Equine sleep can also be greatly affected by changes within the environment (Greening et al., 2021) with evidence of sleep disturbances occurring within EEG profiles (Kalus, 2014). In this context, the horse may be a useful model for the study of sleep quality in a non-human species. Therefore, the aim of this study was to develop a behaviour-based measurement of sleep quality in the horse, which could be used more broadly as a methodological approach for other non-human species.

## RESULTS

The following presents significant results for each of the five indices. Non-significant model outputs can also be found in Table S1.

### Total SQI

**Fig. 1.**
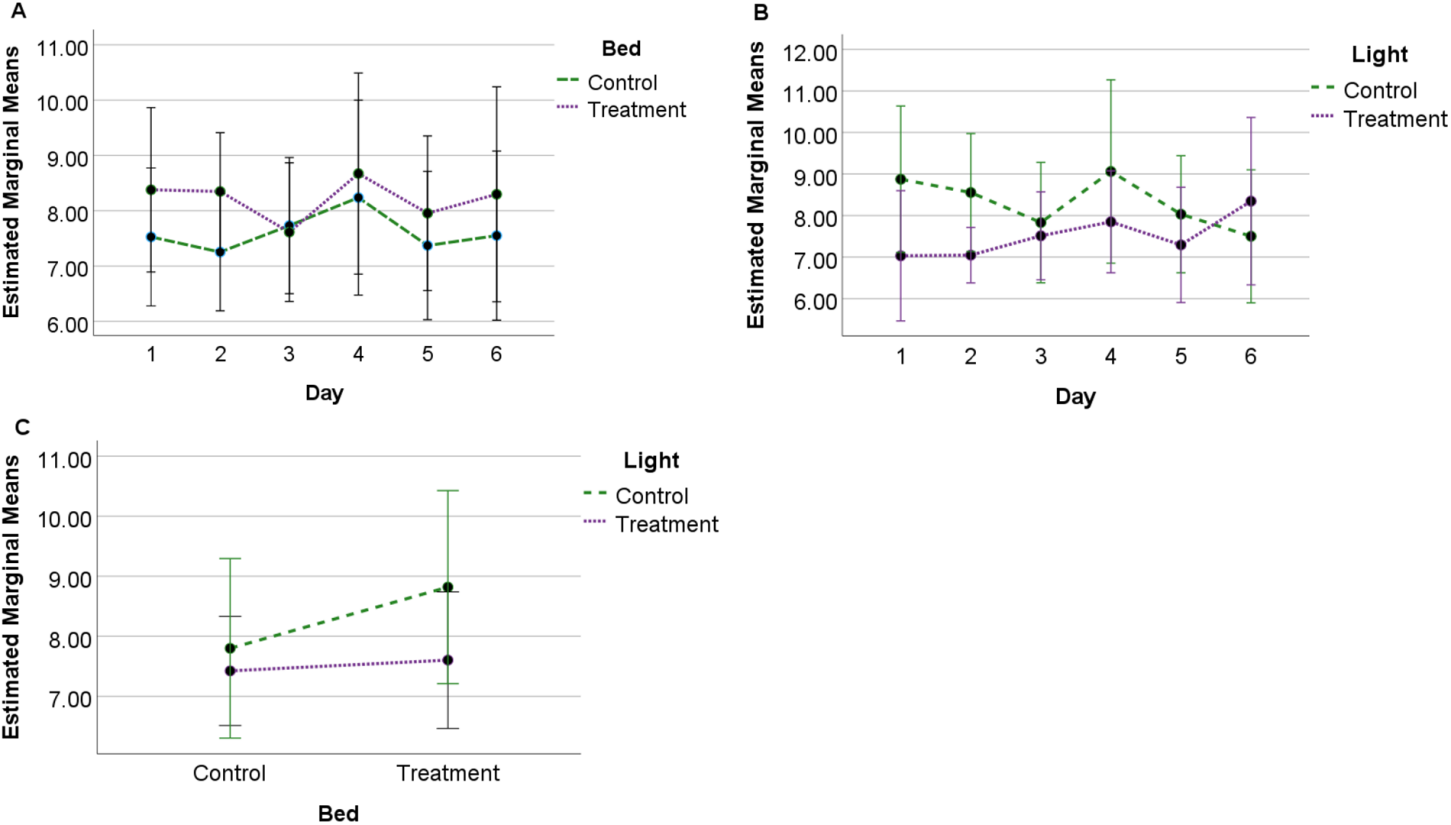
**Estimated marginal means (standard deviation, s.d.) for total SQI score.** Observed for 24 h across 6 days under each experimental condition (*N*=10) showing (A) profile of SQI scores for bedding over 6 days; (B) profile of SQI scores for lighting over 6 days, and (C) interaction between bedding and lighting conditions.

A significant difference (*F*=4.942, *P*=0.05, η^2^=0.354) in total SQI was observed between control bedding [mean 7.61, standard error (s.e.)=0.55] and treatment bedding conditions (mean 8.21, s.e.=0.66), but not between control-light and treatment-light conditions. No significant interactions were observed between bed and light or bed/light and day. No significant effects were observed for lighting or bedding conditions between days (Fig. 1).

### Combined SQI

**Fig. 2.**
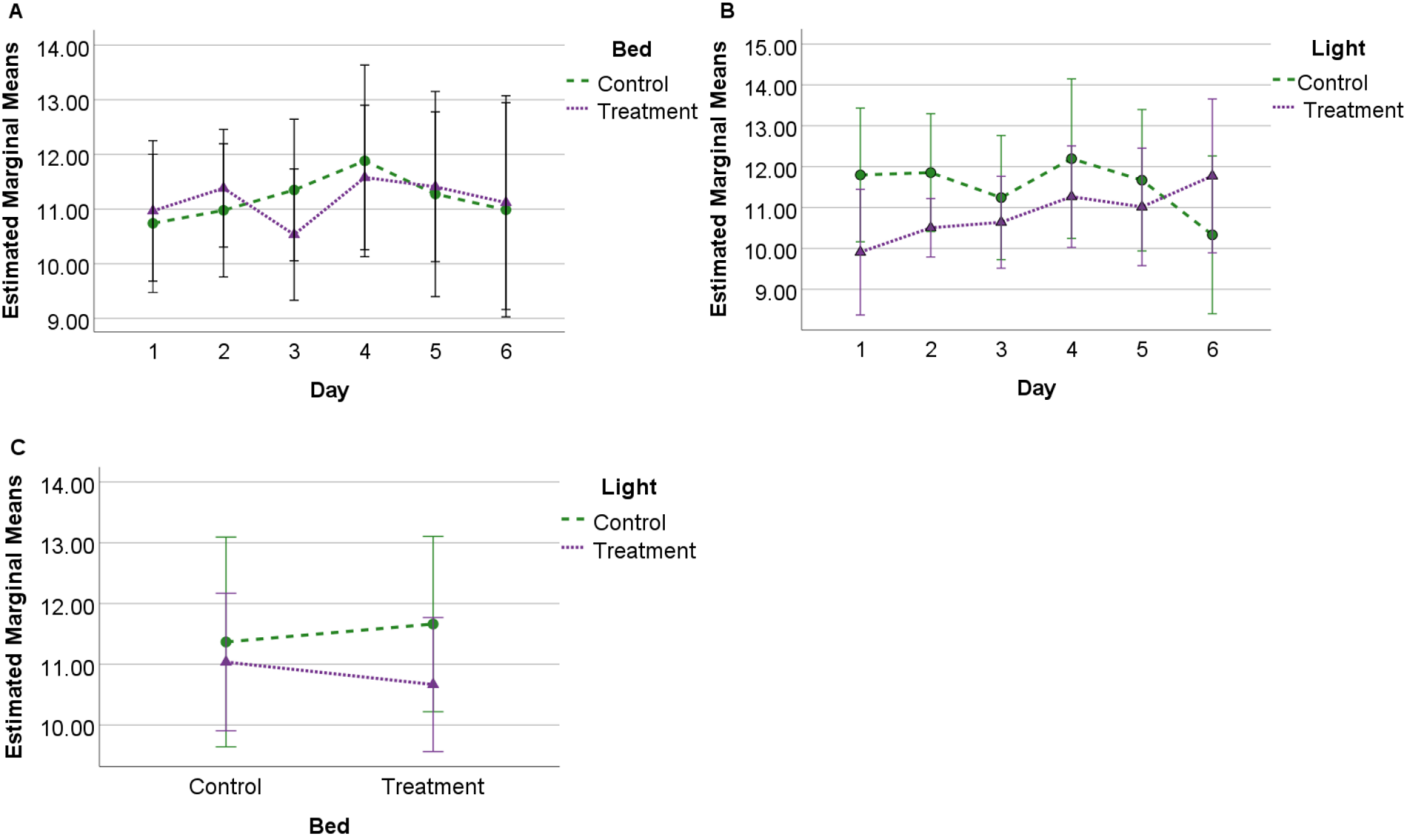
**Estimated marginal means (s.d.) for combined SQI score.** Observed for 24 h across 6 days under each experimental condition (*N*=10) showing (A) profile of SQI scores for bedding over 6 days; (B) profile of SQI scores for lighting over 6 days, and (C) interaction between bedding and lighting conditions.

There were no significant differences between treatment or control conditions for lighting or bedding, nor any significant interactions between bed and light. A significant interaction of lighting with day (*F*=3.674; *P*=0.007, η^2^=0.290) was observed between light conditions. Post-hoc analysis found a significant difference (*P*=0.048) between control light (mean 11.85, s.e.=0.72) and treatment light (mean 10.50, s.e.=0.36) on day two. No significant interactions with day was detected for different bedding conditions, or for interactions between days for bedding or light (Fig. 2).

### Combined weighted SQI

**Fig. 3.**
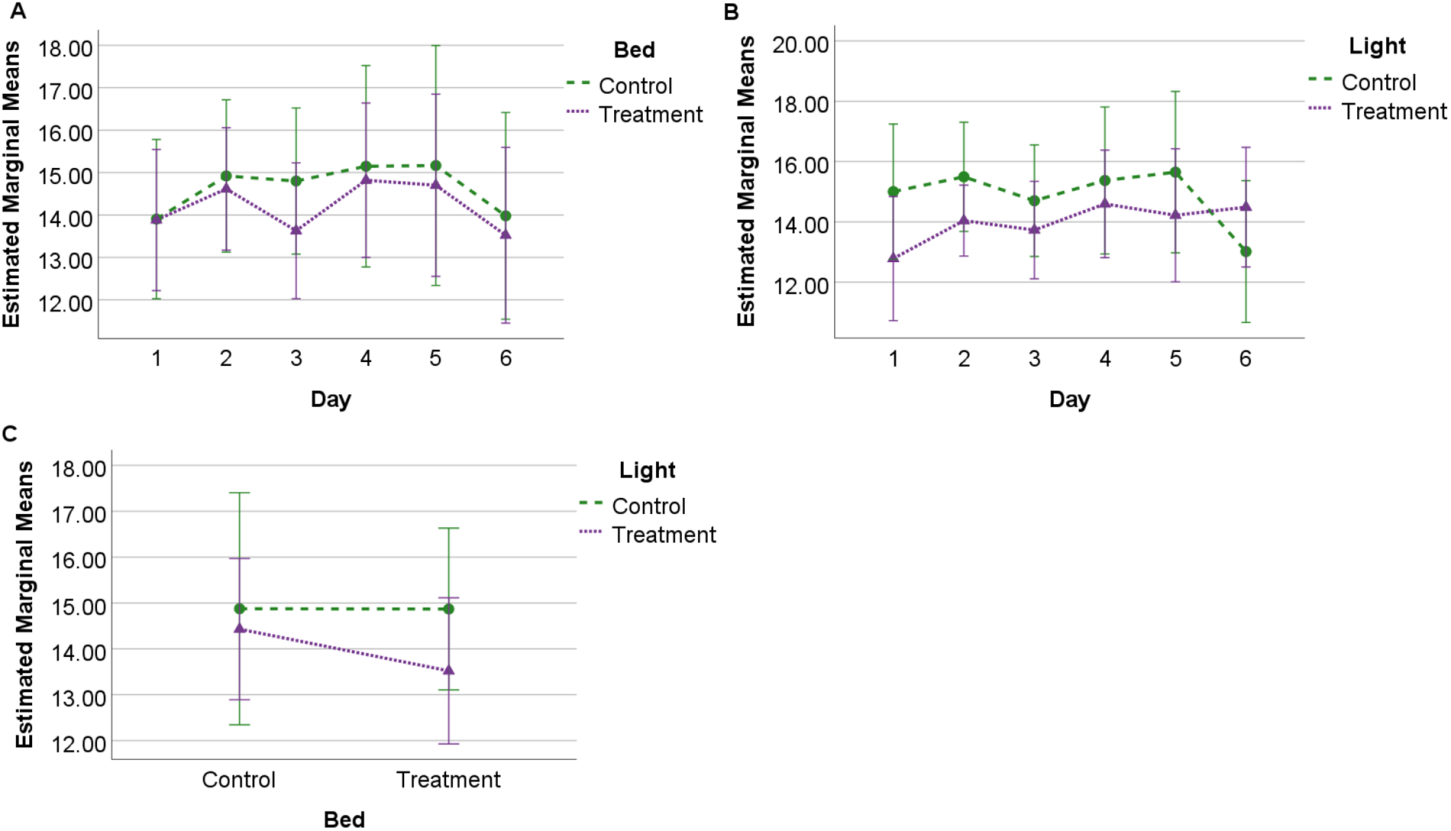
**Estimated marginal means (s.d.) for combined weighted SQI score.** Observed for 24 h across 6 days under each experimental condition (*N*=10) showing (A) profile of SQI scores for bedding over 6 days; (B) profile of SQI scores for lighting over 6 days, and (C) interaction between bedding and lighting conditions.

There were no significant differences for treatment or control conditions for lighting or bedding, nor any significant interactions between bedding and light. A significant interaction between days for control light (*F*=2.477, *P*=0.046, η^2^=0.216) was observed. Post-hoc analysis found a significant difference (*P*=0.034) between day two (mean 15.49, s.e.=0.91) and day six (mean 13.02, s.e.=1.18) (Fig. 3). There were no significant differences between days for treatment lighting, or control and treatment bedding conditions, nor were there significant interactions with day for lighting and bedding conditions.

### REM and NREM SQI

**Fig. 4.**
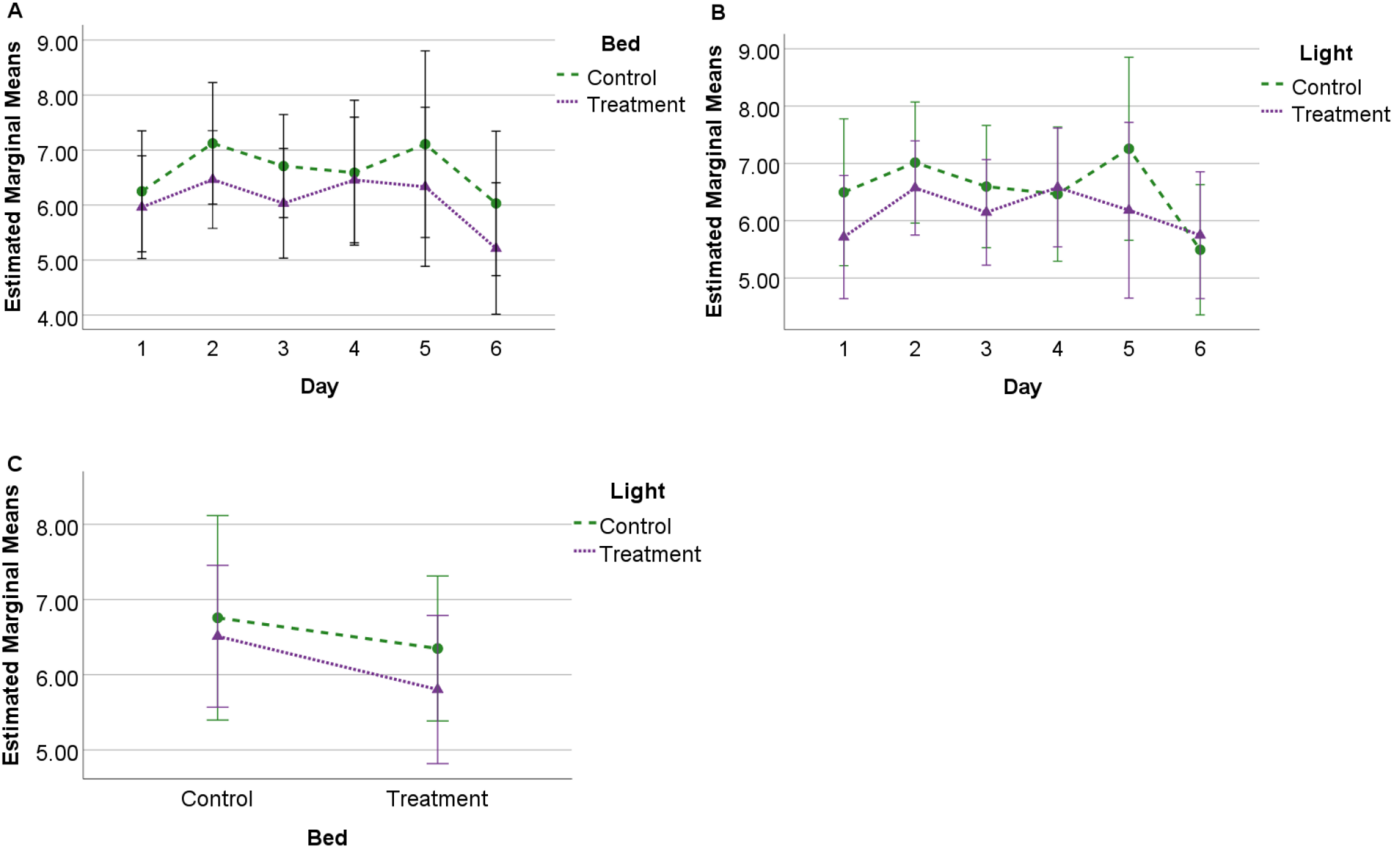
**Estimated marginal means (s.d.) for REM SQI score.** Observed for 24 h across 6 days under each experimental condition (*N*=10) showing (A) profile of SQI scores for bedding over 6 days; (B) profile of SQI scores for lighting over 6 days, and (C) interaction between bedding and lighting conditions.

**Fig. 5.**
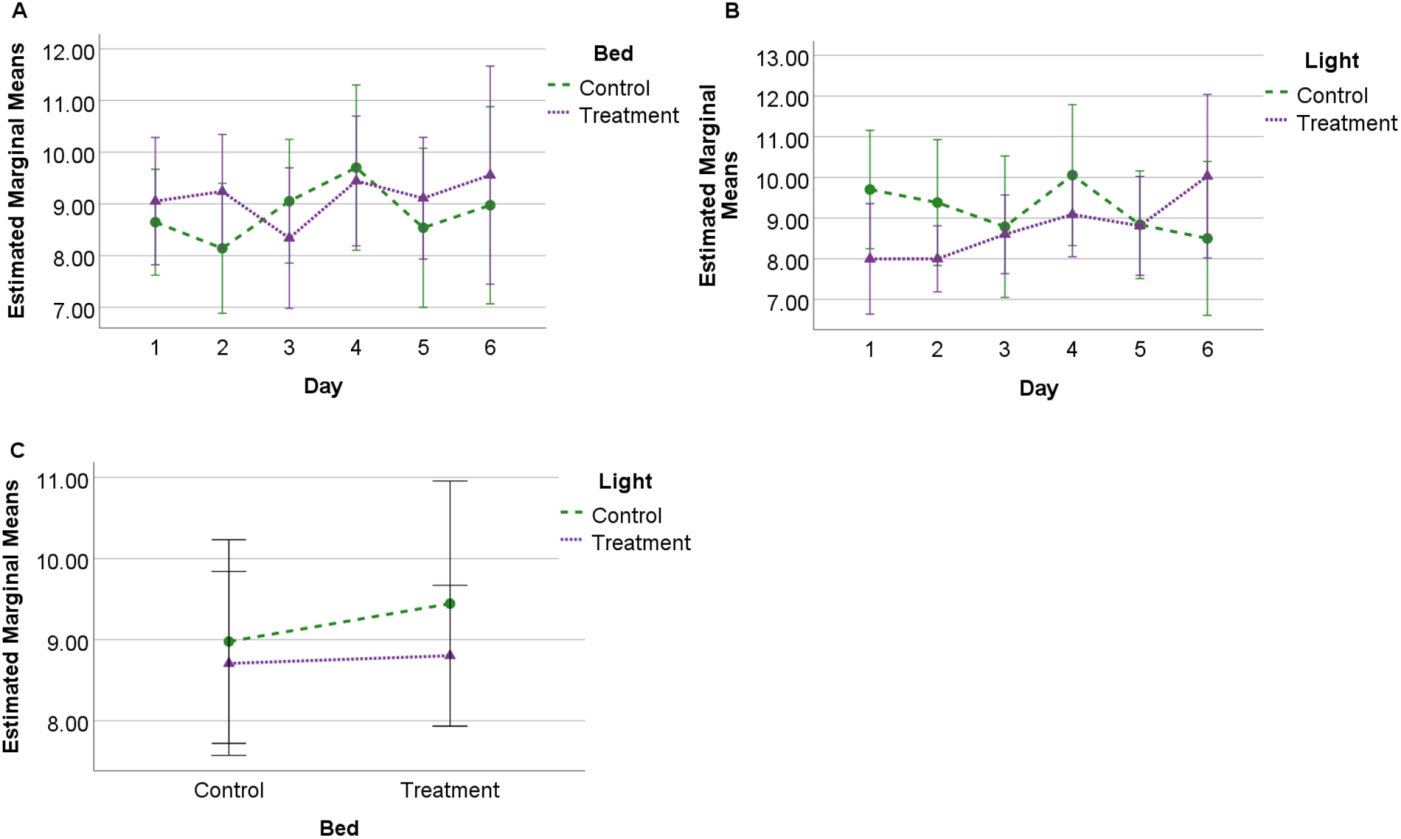
**Estimated marginal means (s.d.) for NREM SQI score.** Observed for 24 h across 6 days under each experimental condition (*N*=10) showing (A) profile of SQI scores for bedding over 6 days (B) profile of SQI scores for lighting over 6 days, and (C) interaction between bedding and lighting conditions.

No significant differences between scores were reported for any variable for the REM SQI calculation (Fig. 4). For the NREM SQI, there were no significant differences between treatment or control conditions for lighting or bedding, nor any significant interactions between bed and light. A significant effect of day (*F*=3.83, *P*<0.01, η^2^=0.30) was observed between light conditions. Post-hoc analysis found a significant difference (*P*=0.02) between control light (9.38, s.e.=0.77) and treatment light (7.99*, s.e.=0.41) on day two (Fig. 5). There were no significant differences between days for each bedding and lighting condition.

## DISCUSSION

Total sleep SQI scores were significantly lower for control (deeper bed) compared to treatment (thinner bed) bedding conditions, suggesting that thinner beds encouraged greater quality sleep through reduced sleep disturbance. Comparatively, the duration of sleep behaviour was greater for standing NREM sleep under the treatment bedding conditions, with less time spent in sternal recumbency (NREM sleep) and lateral recumbency (REM sleep) during the treatment bedding (Greening et al., 2021). In this context, it is possible that sleep is more disturbed when horses are in recumbent postures, so that when this type of sleep increases in duration (as during the control bedding) it is also susceptible to greater interruption. However, when REM SQI was calculated by itself, scores tended to be higher on control bedding (although these findings were not significant, Fig. 4). Scores also tended to be higher for the combined weighted SQI and REM SQI on control bedding. Although these findings were also not significant, they suggest that fundamental differences exist between the quantity and quality metrics of sleep that require further investigation from a functional impact perspective.

Both the duration of sternally recumbent REM sleep (Greening et al., 2021) and the combined SQI and NREM SQI (on day two) scores were significantly lower during the treatment light versus control conditions. Results suggest that not only were horses engaging in significantly less sternal REM sleep but that they were experiencing greater sleep disturbance when the lights were left on overnight. Greening et al., (2021) suggested a pattern of habituation to treatment light within the sleep quantity data, which was also observed for sleep quality except the REM SQI. In this sense, both quality and quantity data appeared to offer reasonable assurance that the behavioural metrics are sensitive to changes in the environment. However, the sleep quality metric appears to be slightly more sensitive to environmental change than duration measurements on a day-by-day basis in that there was a significant interaction of treatment light by day for the combined SQI and NREM SQI but not for the duration data (Greening et al., 2021).

Four indices were derived to enable differentiation between the REM and NREM sleep states and to establish whether these differed from total sleep time. The first was the product of dividing the total amount of sleep with the number of wake episodes, the second was derived from the total time in each sleep state divided by the respective number of wake episodes, and the third index added numerical weight to REM sleep considering the importance of this type of sleep against reduced occurrence compared to NREM sleep. The fourth and fifth indices focused on the number of wake episodes occurring within the total amount of REM and NREM respectively. It is difficult to conclude which index has the best empirical measure of sleep quality given the analyses in this study. REM sleep quality differences were better accounted for within the weighted combined SQI and the separate REM SQI, compared to total SQI, combined SQI, and NREM SQI. The latter indices produced significantly different profiles under the treatment light conditions within this study, suggesting that sleep-quality metrics that give weight to NREM sleep may be more sensitive to changes in environmental condition cues. However, consideration should be given to the association between spontaneous collapse and REM sleep deprivation (Fuchs et al., 2018), where REM-sleep-orientated quality indices may be more useful in this respect. Where the observed horse fails to engage in REM sleep, this, in itself, potentially points at other aspects of reduced welfare (Fuchs et al., 2018). Further work assessing the predictive ability of each of the indices on the impact of sleep disturbance on cognitive and emotional state will help to reveal which index is the most useful from a practical perspective.

While Fuchs et al., (2018) reported high inter- and intra-individual repeatability of total sleep time profiles under standardized conditions, we observed large variation in SQIs between individual horses. This variation in sleep quality is also observed in humans, where, for example, people react differently to the same light exposure (Chellappa, 2021). Further work on animal sleep quality therefore not only needs to establish normal levels of awakenings to determine what constitutes a high and low score within each sleep state for the species in question (Rattenborg et al., 2017), but must also consider inter-individual variation. There is also opportunity to investigate the way in which compensatory mechanisms are employed by individuals, relative to how they cope with environmental changes. For example, NREM stage one sleep is not as restorative as other sleep stages (Brinkman et al., 2023), thus an increase in this sleep stage in conjunction with decreased total sleep time will not sufficiently repay the sleep debt (Wesensten et al., 1999). Future studies also need to investigate the appearance of daytime sleepiness/drowsiness in animals following overnight sleep disturbance, where increased sleepiness is known to increase the likelihood of recovery sleep in humans (Bonnet and Arand, 2003). There is also opportunity to use different objective measurements of sleep quality, such as cumulative wake time through sleep interruptions, and sleep onset latency (SOL) to give further insights into the sleep profile. However, the true validation of both quantity and quality metrics needs to be done in relation to the functional impact of sleep disturbance on the state of the animal. For example, future work using combined metrics of sleep quantity and quality with the horse as a model could assess whether these metrics can both identify the effects of sleep-disturbing factors (such as noise and temperature) and whether these are predictive of changes in the cognitive and emotional state of the animal.

To conclude, changes to the environmental factors of light and bedding depth influenced both sleep quantity and quality. These non-invasive measurements of observable behavioural changes during the sleep state appeared to be useful measures of sleep in a non-human animal species. As an alternative to EEG measurements, this approach is transferable to other animal species but requires that normal patterns for both sleep quality and quantity on a species-by-species basis are established. The treatment-light profiles of both sleep duration and SQIs aligned in response to treatment effects, although SQI appeared to be the more sensitive metric to subtle changes over time. Compared to sleep duration, no significant differences were detected within quality metrics of sleep response to treatment bedding effects, which is fundamentally different from quantity metrics. Sleep quality in different sleep states appeared to differ between the various SQIs, that could also be a consequence of differing environmental cues. Future studies are recommended to consider using REM and NREM SQIs due to the differences between these scores. Further study is also required to test these indices relative to the effects of sleep disturbance from practical and functional (cognitive and emotional) perspectives.

## MATERIALS AND METHODS

### Subjects

Ten horses (six geldings, four mares; mixed breeds; average age 14.9±2.4 years; average height 163.5±7.4 cm, none displaying stereotypic behaviours) had previously been observed on the equine yard at Aberystwyth University via a convenience sampling approach. During the study, subjects were routinely housed in stables (measuring 3.6 m×3.6 m) on the yard for 24 h Monday to Friday then turned out to pasture for 8 h on Saturdays and Sundays, according to their normal routine. As standard practice in all stables, approximately two thirds of the stable floor surface was covered by rubber matting and a straw bed leaving one third as bare concrete that was cleaned as required throughout the day. Horses were accustomed to lights off at 20:00 (after final check), and a straw bedding substrate of 15 cm depth.

### Experimental design

Horses were randomly assigned to two groups balanced for sex and age (group A [*N*=5] or group B [*N*=5]) using the RAND () function in Microsoft Excel, and exposed to two treatment variables (light and bedding) within a two-factor, crossover, repeated-measures experimental design. The control light condition involved the normal turning off (at 20:00) of fluorescent tube lighting (2 lx) overnight, and the treatment light condition maintained the fluorescent tube lighting on (180 lx) during this period. The fluorescent tube lighting was ‘warm white’ with primary spectral peaks of 490 nm, 550 nm and 625 nm. Control bedding involved a bed of normal depth (15 cm), while treatment bedding was maintained at a lower depth (5 cm). Each treatment lasted for 6 days plus a 1-day wash-out period. Groups were staggered in the treatment sequence by 7 days (group B started 7 days earlier and group A finished 7 days later) to facilitate the crossover of experimental lighting conditions, where all the lights were on the same circuit thus either had to be on or off.

High-quality security infrared cameras (Reolink H.264 Digital Video Recorder and ANNKE model N28WEB) were used to record behaviour of all study subjects for 24 h across seven consecutive days during the 5-week study period, according to a predetermined ethogram (Table 2). The study was given ethical approval from the Aberystwyth University Animal Welfare and Ethical Review Board.

**Table 2.**
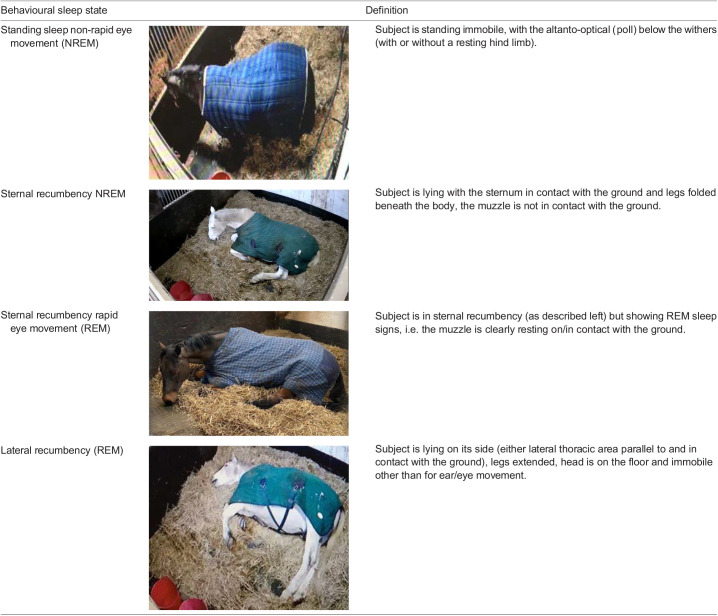
Definition of equine behavioural sleep states (Greening et al., 2021)

### Measuring sleep duration

Sleep behaviour states were designated as being indicative of either REM or NREM sleep states based on behavioural research from previous seminal EEG studies (Greening and McBride, 2022). For example, standing sleep is indicative of NREM sleep, while lateral recumbency was indicative of REM sleep (Table 2). Duration of sleep behaviour was recorded using continuous focal behavioural sampling over a 24 h period for 6 consecutive days per experimental week for each horse. Duration of behaviour was recorded (hours) using continuous focal behavioural sampling over a 24 h period for the 6 days per experimental week for each horse, which was reviewed by three observers. Several training sessions were held to ensure accurate agreement on the sleep behaviours, followed by inter-observer reliability measurement using sample behaviour footage (R2=1).

### Defining sleep interruptions

Greening et al., (2021) had previously recorded behavioural duration data using spreadsheets and the ethogram in Table 2. These data were re-analysed to identify the occurrence of sleep interruptions, defined as awakenings that occurred within any sleep state lasting no more than 3 min (Merica and Gaillard, 1986). An individual horse was considered to have entered a state of wakefulness if an interruption lasted for more than 3 min. For each horse per day, this yielded a frequency count of interruptions lasting <3 min, which were further categorised according to which sleep state they occurred in.

### Development of the sleep quality indices (SQI)

Four different sleep indices were developed using frequency of sleep interruptions, total sleep time and total rapid eye movement (REM) and non-rapid eye movement (NREM) sleep time. For all equations, a higher number of sleep interruptions leads to a lower index score that is indicative of poorer sleep quality.

#### Total SQI

SQI derived from the total sleep time (TST) divided by the number of sleep interruptions using the equation below:
(1)

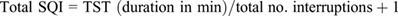
Adding 1 to the number of interruptions eliminated the occurrence of zero data points.

#### Combined SQI

Recognising that different levels of disturbances could occur during different sleep states, the Total SQI equation was adjusted to differentiate data for each behavioural sleep state:
(2)

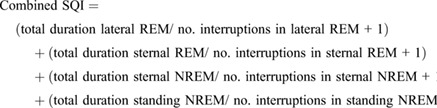


#### Weighted combined SQI

Considering that the horse on average spends approximately 75% of total sleep time in a state of NREM sleep (Greening and McBride, 2022) but that REM sleep may be equally functional from a sleep quality perspective, the second SQI was further adapted to give equal weighting to the REM component of sleep:
(3)

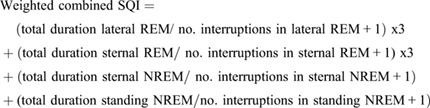


#### REM and NREM SQI

Finally, the different sleep states were subdivided into separate specific equations to determine whether different sleep quality profiles were discernible amongst the two different sleep states.
(4)

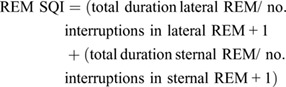

(5)

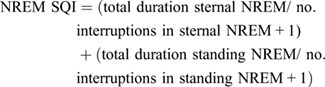


### Data analysis

Each index score was square rooted to normalise data and meet the assumptions for parametric statistical analysis. To assess the effects of light and bedding conditions on sleep quality, index scores calculated over 6 days of each treatment were tested using a two-factor, repeated measures general linear model within SPSS 29. Data are presented using estimated marginal means in order to give the average of the response variable for a factor, adjusted for the means of other factors in the model. Day, bedding and light were set as within-subject factors with no random factor and the model was set at full factorial. Interpolation graphs were generated by SPSS 29, which automatically created error bars with +/– 2 s.e. Post-hoc analysis used Bonferroni (significance set at *P*<0.05). With significant findings, effect size was determined by calculating partial eta squared (large effect η2= >0.14).

## Supplementary Material

10.1242/biolopen.059964_sup1 Supplementary information
