# Supplementary material for: Towards an objective measurement of sleep quality in non-human animals: using the horse as a model species for the creation of sleep quality indices
Source: Biol Open. 2023 Jul 20;12(7):bio059964. doi: 10.1242/bio.059964 (PMC10373578; doi:10.1242/bio.059964)
Supplement: Supplementary information [file biolopen-12-059964-s1.pdf]

Table S1. General linear model output for all indices

Total SQI (assuming sphericity for all factors, except bed*day and light*day using Greenhouse-Geisser)

	df	F	P
Bed	1	4.942	0.053
Light	1	2.977	0.119
Day	5	0.693	0.632
Bed*Light	1	3.264	0.104
Bed*Day	2.269	0.619	0.686
Light*Day	2.663	2.411	0.098
Bed*Light*Day	5	0.925	0.474

Combined SQI (assuming sphericity for all factors, except bed*light*day using Greenhouse-Geisser)

	df	F	P
Bed	1	0.012	0.915
Light	1	1.419	0.264
Day	5	0.856	0.518
Bed*Light	1	1.858	0.206
Bed*Day	5	0.516	0.763
Light*Day	5	3.674	0.007#
Bed*Light*Day	2.06	0.305	0.033#

# denotes significant findings

Combined Weighted SQI (assuming sphericity for all factors)

	df	F	P
Bed	1	0.501	0.497
Light	1	1.306	0.283
Day	5	1.572	0.187
Bed*Light	1	1.593	0.239
Bed*Day	5	0.207	0.958
Light*Day	5	2.477	0.046#
Bed*Light*Day	5	0.430	0.825

# denotes significant findings

REM SQI (assuming sphericity for all factors, except day using Greenhouse-Geisser)

	df	F	P
Bed	1	1.266	0.290
Light	1	0.810	0.392
Day	2.828	2.334	0.101
Bed*Light	1	0.383	0.551
Bed*Day	5	0.264	0.930
Light*Day	5	0.891	0.495

Biology Open • Supplementary information

Bed*Light*Day	5	1.089	0.379

NREM SQI (assuming sphericity for all factors)

	df	F	P
Bed	1	1.407	0.266
Light	1	0.999	0.344
Day	5	0.837	0.531
Bed*Light	1	0.484	0.504
Bed*Day	5	1.029	0.412
Light*Day	5	3.832	0.006#
Bed*Light*Day	5	0.812	0.547

# denotes significant findings

Biology Open • Supplementary information
